# Whole Genome Sequencing Links *Mycobacterium bovis* From Cattle, Cheese and Humans in Baja California, Mexico

**DOI:** 10.3389/fvets.2021.674307

**Published:** 2021-08-03

**Authors:** Alejandro Perera Ortiz, Claudia Perea, Enrique Davalos, Estela Flores Velázquez, Karen Salazar González, Erika Rosas Camacho, Ethel Awilda García Latorre, Citlaltepetl Salinas Lara, Raquel Muñiz Salazar, Doris M. Bravo, Tod P. Stuber, Tyler C. Thacker, Suelee Robbe-Austerman

**Affiliations:** ^1^United States Embassy, U.S. Department of Agriculture, Animal and Plant Health Inspection Service, Mexico City, Mexico; ^2^Programa de Doctorado en Ciencias Quimicobiológicas, Departamento de Inmunología, Escuela Nacional de Ciencias Biológicas, Instituto Politécnico Nacional, Ciudad de México, Mexico; ^3^National Veterinary Services Laboratories, U.S. Department of Agriculture, Animal and Plant Health Inspection Service, Veterinary Services, Ames, IA, United States; ^4^United States Embassy, U.S. Department of Agriculture, Animal and Plant Health Inspection Service, Mexicali, Mexico; ^5^Dirección de Campañas Zoosanitarias de la Dirección General de Salud Animal Servicio Nacional de Sanidad, Inocuidad y Calidad Agroalimentaria, Ciudad de México, Mexico; ^6^Unidad de Investigación, Facultad de Estudios Superiores de Iztacala, Universidad Autónoma Nacional de México, Ciudad de México, Mexico; ^7^Laboratorio de Epidemiología y Ecología Molecular, Escuela Ciencias de la Salud, Universidad Autónoma de Baja California, Ensenada, Baja California, Mexico

**Keywords:** whole genome sequencing, Baja California, bovine tuberculosis, single nucleotide polymorphism, *M. bovis*, cheese

## Abstract

*Mycobacterium bovis* causes tuberculosis (TB) in cattle, which in turn can transmit the pathogen to humans. Tuberculosis in dairy cattle is of particular concern where the consumption of raw milk and dairy products is customary. Baja California (BCA), Mexico, presents high prevalence of TB in both cattle and humans, making it important to investigate the molecular epidemiology of the disease in the region. A long-term study was undertaken to fully characterize the diversity of *M. bovis* genotypes circulating in dairy cattle, cheese and humans in BCA by whole-genome sequencing (WGS). During a 2-year period, 412 granulomatous tissue samples were collected from local abattoirs and 314 cheese samples were purchased from local stores and vendors in BCA and sent to the laboratory for mycobacterial culture, histology, direct PCR and WGS. For tissue samples *M. bovis* was recovered from 86.8%, direct PCR detected 90% and histology confirmed 85.9% as mycobacteriosis-compatible. For cheese, *M. bovis* was recovered from 2.5% and direct PCR detected 6% of the samples. There was good agreement between diagnostic tests. Subsequently, a total of 345 whole-genome SNP sequences were obtained. Phylogenetic analysis grouped these isolates into 10 major clades. SNP analysis revealed putative transmission clusters where the pairwise SNP distance between isolates from different dairies was ≤3 SNP. Also, human and/or cheese isolates were within 8.45 (range 0–17) and 5.8 SNP (range 0–15), respectively, from cattle isolates. Finally, a comparison between the genotypes obtained in this study and those reported previously suggests that the genetic diversity of *M. bovis* in BCA is well-characterized, and can be used to determine if BCA is the likely source of *M. bovis* in humans and cattle in routine epidemiologic investigations and future studies. In conclusion, WGS provided evidence of ongoing local transmission of *M. bovis* among the dairies in this high-TB burden region of BCA, as well as show close relationships between isolates recovered from humans, cheese, and cattle. This confirms the need for a coordinated One Health approach in addressing the elimination of TB in animals and humans. Overall, the study contributes to the knowledge of the molecular epidemiology of *M. bovis* in BCA, providing insight into the pathogen's dynamics in a high prevalence setting.

## Introduction

Bovine tuberculosis (bTB), most commonly caused by *Mycobacterium bovis*, is characterized by the formation of granulomas in the lymph nodes and lungs of infected individuals, though other organs may also be affected (1, 2). It is an OIE (World Organization for Animal Health) reportable disease that infects a broad variety of mammals including humans. Infection in cattle can occur through direct contact by the inhalation of infected aerosols from sick animals and through oral ingestion of contaminated milk, fodder and pastures (3). Humans can acquire the infection also by direct contact with infected animals and through the consumption of contaminated unpasteurized milk and dairy products (4). Due to the significant impact this disease can have on public health and international trade of cattle and their byproducts, programs for the control and eradication of bTB have been implemented in many countries. In developed countries, significant success has been achieved, but wildlife reservoirs have challenged total eradication (5, 6); in least-developed or developing countries, however, the lack of economic compensation for culled animals due to test and slaughter strategies, or the absence of such strategies, complicates control (7, 8).

In Mexico, the bTB National Program classifies geographic territories into two zones (eradication or control) based on the regional bTB herd prevalence over a 12-month period. The program's strategies are to reduce or eliminate the prevalence of the disease in “eradication” zones, which have a bTB prevalence of <0.5%, as well as to prevent reinfection by applying mitigation measures in the movement of cattle from control zones to eradication zones. Eradication zones are primarily populated by beef cattle and currently 86.02% of the country is recognized as being an eradication zone. The bTB prevalence in the remaining control zones (13.98%) is >0.5% (range of 0.1–14.2%) or is unknown and contain primarily dairy cattle (9). The state of Baja California (BCA) is divided into two zones: an eradication zone with <0.5% of bTB herd prevalence and a control zone to the north, which borders California in the US, that is mainly populated by dairy cattle and has reported prevalence rates as high as 40% (10). As previously reported in the literature (11), there is very high prevalence of tuberculosis in cattle and humans in BCA and although *M. tuberculosis* is the main causative agent of TB in humans, *M. bovis* may play an important role in areas where bTB is endemic and even more so where bTB prevalence rates in cattle are high (12). In Mexico there is limited information available with respect to human TB caused by *M. bovis* and some studies have reported a median percentage of 7.6% (range 0–31.6%) (13, 14). However, due to a lack of species identification in the diagnosis, cases of bTB in humans are likely underestimated (15). Additionally, in the US, 90% of human bTB cases are usually traced to people of Hispanic communities, most of which have origins in Mexico (16). Interestingly, San Diego County in California is the only county in the US which borders a control zone and reports the highest levels of *M. bovis* infection in people (17, 18). Consequently, this high TB burden dairy region of BCA may be contributing to that high level of *M. bovis* detection. Furthermore, isolation of *M. bovis* from cheese has been reported (19) and the association of human bTB cases in Hispanic people from Mexico has been attributed to the consumption of contaminated cheese produced with unpasteurized milk (20). The production of artisanal cheese is customary in Mexico and is often carried out by traditional “cheese-makers” in the rural areas of the country, to be later sold at the open markets and small stores (21). Comparably, cases of human bTB in the US have also been associated to fresh cheese brought into the US from Mexico (22).

Recovery of *M. bovis* from raw milk and cheese is challenging and relies on decontamination methods that can maintain the delicate balance between inactivation of undesirable microorganisms and the viability of mycobacteria, thus appropriate processing procedures such as homogenization, decontamination, concentration and culture media, must be selected to facilitate optimum recovery of mycobacteria (23). Previous studies at the National Veterinary Services Laboratories (NVSL) have evaluated the isolation of *M. bovis* and *M. avium* subsp. *paratuberculosis* from milk and cheese using various combinations of decontamination processes and have eventually settled on a chemical combination of N-acetyl-l-cysteine–sodium hydroxide (NALC-NaOH) for decontamination (19, 24, 25). Furthermore, comparisons of different types of media for the optimal recovery of *M. bovis* have yielded the best results for 7H11P (Middlebrook 7H11 agar supplemented with sodium pyruvate, calf serum, lysed sheep blood and malachite green) and BACTEC MGIT 960 (Becton Dickinson Diagnostic Systems, Sparks, MD) supplemented with an antibiotic mixture (BBL MGIT PANTA, Becton Dickinson) (26–28).

In contrast to the bTB prevalence in BCA dairy cattle, the US cattle herd has a very low prevalence of bTB (<0.001%) and several studies have suggested most new cattle herd detections are the result of new introductions and not continued spread within local cattle (29, 30). While many sources of bTB introduction remain unknown, some of the genotypes have closely matched isolates recovered from previous studies in BCA (11). Since dairy cattle movements are strictly controlled in Mexico, humans and fomites may be a likely source as this region serves as a major port of transit for people and goods, including fresh cheese. To address this issue, a binational collaboration was initiated with three overall objectives: (1) fully characterize the diversity of *M. bovis* genotypes circulating in BCA by whole-genome sequencing, (2) to determine the role of fresh cheese from the region as a potential source of infection to humans and (3) compare the genotypes identified in BCA to those previously reported for the region, the rest of Mexico and the US.

## Materials and Methods

### Study Area

**Figure 1:**
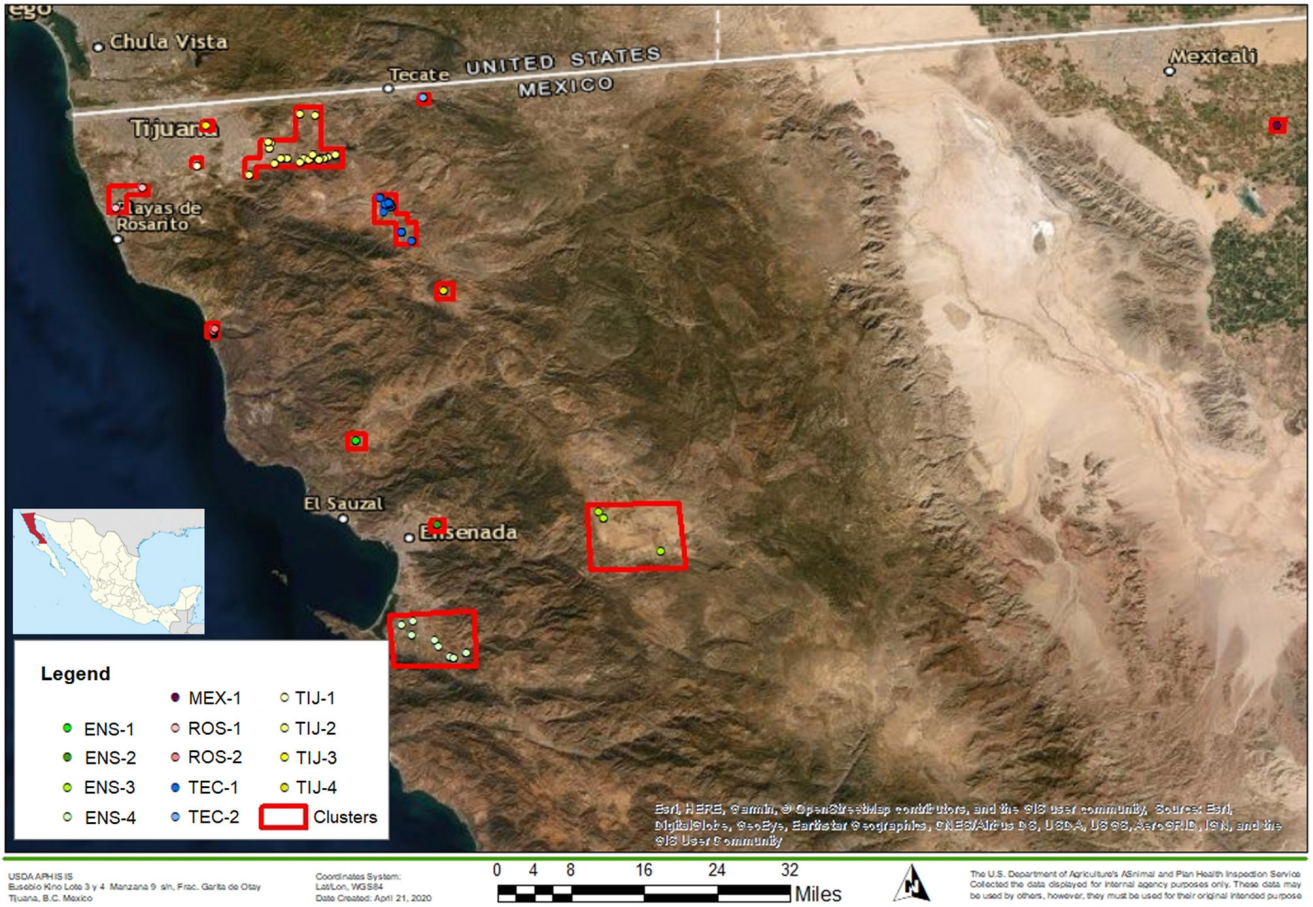
Map of the northern region of Baja California. The location of BCA with respect to Mexico is shown on the left. Red squares represent dairy clusters (*n* = 13) formed based on a maximum distance of 5 km (radius) between dairies. Clusters' names are indicated in the legend. The map was built using ArcGIS software (basemap WGS1984).

The study was performed in the control zone of Baja California, Mexico, which is the dairy region located in the far northwest of the state (Figure 1). The location of the dairy herds from which the sampled cattle originated was mapped and clusters were formed based on a maximum distance (radius) of 5 km between the dairies. Thirteen clusters were assigned as follows: Ensenada 1 (ENS-1), Ensenada 2 (ENS-2), Ensenada 3 (ENS-3), Ensenada 4 (ENS-4), Mexicali 1 (MEX-1), Rosarito 1 (ROS-1), Rosarito 2 (ROS-2), Tecate 1 (TEC-1), Tecate 2 (TEC-2), Tijuana 1 (TIJ-1), Tijuana 2 (TIJ-2), Tijuana 3 (TIJ-3) and Tijuana 4 (TIJ-4). A full list of the dairies that corresponded to each cluster is in Supplementary File 1.

### Tissue Samples

From October 2016 to November 2018, tissue samples with bTB-suspicious lesions (granulomas) were collected by an accredited veterinarian during post-mortem inspection of dairy cull cattle, under the supervision of the abattoir's official veterinarian. Three abattoirs, each in the municipalities of Ensenada, Tecate and Tijuana, were targeted. Based on estimations by the Mexican Secretariat of Agriculture and Rural Development (Secretaría de Agricultura y Desarrollo Rural, SADER), these three abattoirs receive over 90% of the cull dairy cows in the region. The abattoirs were visited 2–3 times per week on days in which the volume of animals was highest. Each sample was divided in two: one half was stored in formalin for histopathological analysis and the second half was frozen for bacteriological analysis. Epidemiological data associated to each animal's official identification tag (SINIIGA) was collected, such as owner, farm of origin (location, production unit), dealer, transit document number (movement authorization), etc. (https://www.siniiga.org.mx/identifica.html). Finally, samples were submitted to the USDA's National Veterinary Services Laboratories (NVSL) in Ames, Iowa for analysis.

### Cheese Samples

Fresh cheese samples were collected throughout the region of Ensenada, Rosarito, Tecate and Tijuana in BCA from October 2016 to December 2019. Approximately 250 g pieces were purchased from informal sellers, small stores and markets, 2–4 times per week. Samples were stored in sterile, airtight containers and shipped in styrofoam coolers with icepacks to NVSL for analysis.

### Mycobacterial Isolation and Identification

#### Tissues

Prior to culture, a pea-sized sample was obtained for direct PCR. Then, granuloma samples were trimmed of excess fat and connective tissue and soaked for 20−30 min in a 1:100 solution of bleach and R/O water, then tissues were homogenized. Seven mL of macerated tissue were placed in 5 mL of 1 N NaOH and decontaminated for 7–10 min and neutralized to effect with the MycoDDR Neutralization Buffer B (Immuno-Mycologics, Inc., US) to a final volume of 35 ± 5 mL. Specimens were centrifuged at 4,700x*g* for 25 ± 2 min at 10°C and the supernatant decanted off. Pellet was resuspended in 2–3 mL of PBS and was inoculated into BACTEC MGIT 960 (Becton Dickinson, Sparks, Md.) for up to 42 days and two tubes of Middlebrook 7H11 media with sodium pyruvate and incubated at 37°C for up to 8 weeks.

#### Cheese

Mycobacterial isolation from cheese samples was performed following a previously described methodology (19). Briefly, 5 g portions of cheese were weighed and aseptically transferred into a blender jar containing 45 mL of 2% sodium citrate. The cheese was homogenized and the jars were placed in a 37°C water bath for 1 h to help liquefy the specimen. The cheese suspension was decontaminated using the N-acetyl-L-cysteine (NALC)-NaOH method (31). 10 mL of the liquefied and homogenized sample was mixed with 10 mL of digestant containing NaOH-Sodium citrate and NALC. The mixture was allowed to stand at room temperature for 15–20 min. 30 mL of phosphate buffer was then added. The mixture was then centrifuged at 4,700x*g* for 25 min at 10°C and the supernatant decanted off. Pellet was resuspended in 2–3 mL of PBS and was inoculated into BACTEC MGIT 960 (Becton Dickinson, Sparks, Md.) and incubated for up to 42 days and two tubes of Middlebrook 7H11 media with sodium pyruvate (7H11P) and incubated at 37°C for up to 8 weeks.

#### Isolate Identification by PCR and Sanger Sequencing

Real-time PCR against *IS*1081was performed on DNA extracted from acid fast colonies either from solid media or MGIT media. If the Ct value was below 14, the DNA was sent for whole genome sequencing. If the PCR was above 14, the isolate was subcultured on to fresh 7H11P solid media and allowed to grow. If the PCR was negative, the DNA was sent for Sanger sequencing using both universal primers against 16S rDNA and mycobacterial specific primers for rpoB and the sequences were blasted against GenBank.

### Direct PCR

Direct real-time PCR was performed directly from tissue as previously described (32), with modifications. Direct PCR was performed on a pea-sized sample obtained from the tissue used for culture. Briefly, tissues were examined for granulomatous lesions and dissected to obtain a pea-sized subsample. Subsamples were transferred into 2 mL screw-cap microcentrifuge tubes with a glass bead mixture of approximately 125 μL of 1.0 mm and 125 μL of 0.1 mm beads. 400 μL of a buffer solution containing approximately 400 μL 1X TE buffer and 2.5 μL of DNA Extraction Control 670 were added per sample. Samples were heat-inactivated in a heat-block at 100°C for 30 min and posteriorly bead-disrupted at full speed for 2 min using a mini-bead beater. Samples were then centrifuged at 16,000x*g* for 5 min. The top aqueous layer was used to extract mycobacterial DNA using the MagMax CORE nucleic acid purification kit (Applied Biosystems, ThermoFisher Scientific, US) and a KingFisher Flex System (Thermo Fisher Scientific, Waltham, MA, USA). Real time PCR was performed on the QuantStudio or Viia7 instruments (Applied Biosystems, California, USA).

For cheese, 400 μL of the homogenate were transferred into 2 mL screw-cap microcentrifuge tubes with a glass bead mixture of approximately 125 μL of 1.0 mm and 125 μL of 0.1 mm beads. PCR tubes contained a total volume of 500 μL: 98.5 μL of 1X TE buffer, 2.5 μL of DNA Extraction Control 670 and 400 μL of phenol/chloroform. After inactivation with the phenol/chloroform, samples were bead-disrupted and processed the same as for tissue as mentioned above.

### Whole Genome Sequencing and Data Analysis

DNA from colonies was extracted using the MagMax CORE nucleic acid purification kit (Applied Biosystems, ThermoFisher Scientific, US) and a KingFisher Flex System (Thermo Fisher Scientific, Waltham, MA. USA). Real time PCR was performed on the QuantStudio or Viia7 instruments (Applied Biosystems, California, USA). A minimum of 20 μL of DNA sample with a minimum concentration of 5 ng/μL was required for sequencing. Sequencing was performed in an Illumina MiSeq device (Illumina, San Diego, CA, USA), according to manufacturer's instructions, using 250 bp paired-end read chemistry and libraries were prepared using the Nextera XT Library Preparation Kit (Illumina, San Diego, CA, USA) also according to manufacturer's instructions. Raw FASTQ files were analyzed with the vSNP pipeline (https://github.com/USDA-VS/vSNP). Quality check is done as part of the vSNP package (see Supplementary File 5 for sequencing metrics). Briefly, FASTQ files were used to align reads against the reference genome *M. bovis* AF2122/97 (NCBI RefSeq Accession NC_002945.4) using BWA-mem (33). 80X depth of coverage was targeted. SNPs were called using FreeBayes (34) and visually validated with IGV (35). Phylogenetic trees were constructed based on whole genome concatenated SNP sequences using RAxML (36) under a GTR-CAT model of substitution. Tree visualization, annotation and editing was performed with FigTree (37). As output from the vSNP pipeline, SNP tables for each major clade were generated; these are formatted Excel tables that group and sort isolates and SNP according to relatedness and reflect exactly what is shown by the phylogenetic tree, which provides transparency of the results. In the SNP tables the columns identify the genome location of the SNP calls and the isolates are listed in the rows. The reference (*M. bovis* AF2122/97, NC_002945.4) is listed across the top and is identified as the “reference call.” All SNP are highlighted. Map-quality for each SNP is indicated and is an average of the map quality scores of each isolate at that position. A score of 60 is the highest possible. Finally, the annotation of the position is listed at the bottom of the SNP table.

The complete analysis involved the sequences generated in this study and sequences from the NVSL database generated by previous studies (11, 30, 38), which are publicly available in the NCBI Sequence Read Archive under Bioprojects PRJNA384996, PRJNA251692 and PRJNA449507, respectively (Supplementary File 4).

#### Cluster Analysis

A cut-off value for pairwise SNP distances between isolates of 12 SNP has been widely used for *M. tuberculosis* transmission studies (39–41). Here, a cut-off value of 10 SNP was used based on patterns observed in the data by the careful visual inspection of the SNP matrices (Excel tables) obtained from the vSNP pipeline. Each cluster was identified and labeled based on the defining SNP for that cluster according to its genome position with respect to the reference genome (*M. bovis* AF2122/97, RefSeq accession number NC002945.4). Additionally, a cut-off value of 3 SNP was also used for identifying putative transmission between the dairies.

### Statistical Analysis

To evaluate concordance between laboratory tests for the diagnosis of *M. bovis* (culture, histology and PCR), Cohen's kappa coefficient was determined for histology and culture and for PCR and culture, as culture remains the gold standard for *M. bovis* confirmatory diagnosis. The following formula was applied:

(1) $$\begin{array}{r} K=\left(Po-Pe\right)/\left(1-Pe\right) \end{array}$$

where Po refers to the observed agreement between tests and Pe refers to the expected agreement. For this, Pe was determined with the following formula:

(2) $$\begin{array}{r} Pe=\left[\left(n1/n\right)\;*\;\left(m1/n\right)\right]\;+\;\left[\left(n0/n\right)\;*\;\left(m0/n\right)\right] \end{array}$$

where the values n1, n, m1, n0 and m0 are based on the following:

**Table:** 

**Histology/PCR**	**Culture Positive**	**Negative**	**Total**
Positive	a	b	m_1_
Negative	C	d	m_0_
Total	n_1_	n_0_	n

Cohen suggested the Kappa result be interpreted as follows: values ≤ 0 as indicating no agreement and 0.01–0.20 as none to slight, 0.21–0.40 as fair, 0.41–0.60 as moderate, 0.61–0.80 as substantial and 0.81–1.00 as almost perfect agreement (42).

## Results

### Source Herd Information

Based on cattle census data by Mexico (43), over 95% of the dairies that were larger than 100 head were sampled during the study. Animals were housed in open-air dry-lot dairies where modern management practices are in place, similar to what can be seen in developed countries such as the US. The main breeds in these dairies were Holstein and Swedish Red, with an average milk production of 25 liters per day. This region of the country is an ideal environment for dairy production and upon observation, the animals appear healthy and with adequate body condition and clinical tuberculosis is not typically recognized.

### Tissue Samples

A total of 445 tissue samples with TB-suspicious lesions were obtained from dairy cattle, which represented a total of 90 dairies. Unfortunately, 33 samples failed to make it to the laboratory in acceptable condition for testing due to shipping logistics. Consequently, 412 samples were included in the analysis, representing a final total of 61 dairies. Ten samples could not be traced back to a dairy, so they were labeled as “Unknown.” Of the total, 363 (88.1%) samples corresponded to lymph nodes (superficial cervical, mandibular, parotid, retropharyngeal, tracheobronchial, mediastinal and hepatic), 10 were from lung (2.4%) and 39 from liver (9.5%). The number of granulomas collected per dairy varied widely between 0 and 35. The overall detection rate for *M. bovis* from tissue samples was 86.9%, with lymph nodes achieving the highest rate at 91.7% (Table 1).

**Table 1:** Total tissue samples obtained from dairy cattle in Baja California, Mexico for the detection of *M. bovis*.

**Type of tissue**	**Total number of samples**	**Samples detected with *M. bovis***	**Proportion detected (%)**
Lymph nodes	363	333	91.7
Superficial cervical	1	1	100.0
Hepatic	12	12	100.0
Mandibular	8	6	75.0
Mediastinal	24	21	87.5
Parotid	12	12	100.0
Retropharyngeal	217	196	90.3
Tracheobronchial	89	85	95.5
Lung	10	6	60.0
Liver	39	19	48.7
Total	412	358	86.9

### Cheese Samples

A total of 314 cheese samples were included in the analysis (Supplementary File 6). Overall, 22 (7%) were reported out as contaminated (overgrowth of non-acid fast bacteria); 262 (83.4%) were reported as no isolation made, and 30 (9.6%) contained acid fast bacteria, of which 8 (2.5%) were *M. bovis*. Other acid fast bacteria recovered from the cheese included 17 *M. porcinum* isolates, and one each of *M. bolletii, M. fortuitum and M. neoaurum*; and two atypical, likely unnamed mycobacteria with sequences that did not match close enough for species determination.

A total of 85 stores were visited throughout the four municipalities. *M. bovis* was cultured from all four municipalities, one store in Tecate, two different stores in Rosarito, one store in Tijuana (two samples collected at different times), and one store in Ensenada (three samples collected at different times). Even though the number of samples for “fresh” cheese (188) was greater than “panela” cheese (126), the isolation rate of *M. bovis* by culture was the same for both (2.7 and 2.4%, respectively). Similarly, the detection rate of *M. bovis* from fresh and panela cheese by direct PCR was not significantly different between the two types, having obtained detection rates of 5.8% (11/188) and 6.3% (8/126), respectively. Overall, the detection rate was higher for direct PCR (5.7%) compared to culture (2.5%). All the *M. bovis* isolates recovered from these cheese samples had different WGS sequences, suggesting different cow sources despite several obtained from the same store.

### Culture, Histology, and Direct PCR

For *M. bovis* detection, cattle tissues were processed by direct PCR, culture and histology. A comparison of culture, histology and direct PCR results is shown in Table 2. Of the 412 tissue samples, 358 were positive for culture, 354 were positive for histology (mycobacteriosis-compatible) and 345 were detected by both. Direct PCR alone detected 371 samples and 351 were detected by both PCR and culture. Six culture-positive samples were not detected by direct PCR and 20 culture-negative were detected by direct PCR. For culture and PCR, the kappa obtained was 0.6774 (CI 95% 0.6398–0.7149), indicating substantial agreement between these tests. For histology and culture, 345 samples were detected by both tests, nine were “mycobacteriosis compatible” but culture-negative and 13 identified as “other diagnosis” by histology were positive for culture. Thereby, a kappa statistic of 0.7727 (CI 95% 0.7449–0.8004) was obtained for culture and histology, also indicating a substantial level of concordance between the two tests (Table 2).

**Table 2:** Distribution of culture, histology and direct PCR results for the detection of *M. bovis* from tissue from dairy cattle from Baja California, Mexico.

	**Culture**		
**Direct PCR**	**Positive**	**Negative**	**Total**
Detected	351	20	371
Not Detected	6	34	40
Inconclusive	1	0	1
Total	358	54	412
**Histology**
Mycobacteriosis compatible	345	9	354
Other diagnosis	13	45	58
Total	358	54	412

Additionally, Table 3 details the various diagnosis classified as “other diagnosis,” representing the samples identified as negative by histology. Thirteen samples fell under this classification and included eosinophilic granuloma (*n* = 1), granuloma of unknown etiology (*n* = 2), lymphoid hyperplasia (*n* = 1), lymphoplasmitic hepatitis (*n* = 1), pyogranuloma (*n* = 6) and no significant findings (*n* = 2).

**Table 3:** Comparison between histology and culture results for the detection of *M. bovis* from granulomas obtained from dairy cattle in Baja California, Mexico.

	**Culture results**		
	***M. bovis* isolated**	**No isolation**	**Total**
**Histology diagnosis**
Mycobacteriosis-compatible	345	9	354
Abscess	0	4	4
Actinobacillosis or mycosis	0	9	9
Chronic pneumonia	0	2	2
Coccidioidomycosis	0	2	2
Eosinophilic granuloma	1	0	1
Granuloma, unknown etiology	2	1	3
Hepatitis	0	1	1
Lymphoid hyperplasia	1	1	2
Lymphoplasmitic hepatitis	1	0	1
Lymphosarcoma	0	2	2
Microgranuloma	0	1	1
Mycotic granuloma	0	1	1
No significant findings	2	5	7
Pyogranuloma	6	16	22
Total	358	54	412

### WGS and SNP Clusters

**Figure 2:**
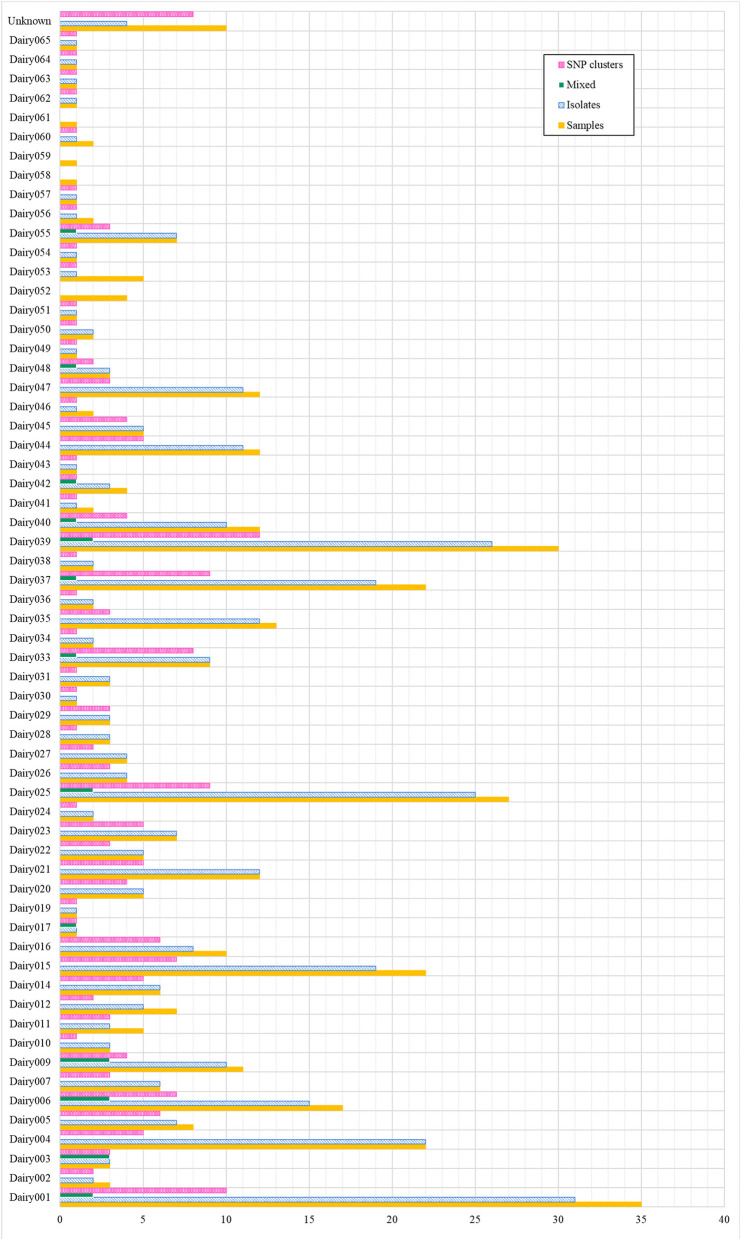
Baja California dairies with positive detection of *M. bovis*. For each dairy, the figure indicates the total number of samples, isolates and SNP clusters. “Mixed” refers to isolates that had a high proportion (≥50%) of ambiguous SNP calls, indicating the presence of more than one genotype. “Unknown” refers to dairies that were not possible to trace back.

Of the 61 final total dairies, 58 (93.5%) were confirmed to contain animals infected with *M. bovis*, having obtained at least one isolate from an animal in the dairy (Figure 2). The highest number of isolates obtained from a single dairy was 31 (Dairy001) and a single isolate was recovered for 17 dairies. Also, at least one and up to 12 SNP clusters were identified for a single dairy (Dairy039), only Dairy053 and Dairy059 were not associated to a SNP cluster. Additionally, 20 isolates that corresponded to 13 dairies were indicative of mixed infection due to their high proportion of ambiguous calls (≥50%), which suggests the presence of more than one genotype.

**Figure 3:**
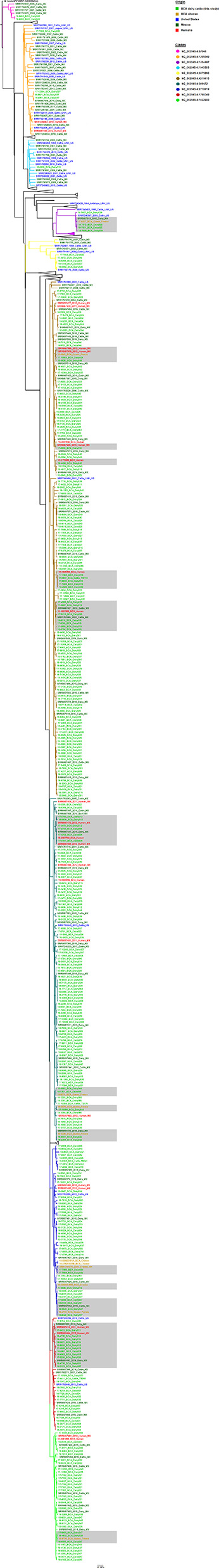
Whole-genome SNP-based phylogeny of 642 *M. bovis* isolates obtained from cattle, cheese and humans from Baja California, Mexico (BCA) and the US. The BCA isolates from dairy cattle and cheese obtained from this study correspond to 10 main clades, as indicated in the legend. Each clade is labeled according to the genome position of the defining SNP for that clade, with respect to the reference genome *M. bovis* AF2122/97 (NC002945.4). Gray-shaded areas indicate clusters of human and/or cheese isolates that are within 10 SNPs of a recent common shared ancestor. Some clades (of unrelated isolates to BCA) were collapsed for to improve visibility. The scale bar represents a distance of 40 SNPs (branch length).

A total of 642 whole genome SNP sequences were included for analysis: 346 from this study (eight from cheese and 338 from dairy cattle) and 297 obtained from GenBank from previous studies (26 human—Mexico, four cheese—Mexico and 267 cattle—from USA and Mexico) (11, 30, 44). Overall, 26 main groups/clades were identified, of which 10 corresponded to the isolates from this study and were labeled according to the defining SNP for each clade (with respect to its genome position in the reference) (Figure 3).

Final alignments (SNP matrices) for each clade are shown in Supplementary File 2. Most of the isolates from this study fell in clades NC_002945.4:4219410 (38%, 132/345) and NC_002945.4:389472 (26%, 90/345). Clades NC_002945.4:57046 and NC_002945.4:1945505 included the least number isolates with 2 and 1, respectively. All the clades included cattle isolates, five clades included cheese isolates (NC_002945.4:389472 = 3; NC_002945.4:2778919 = 5; NC_002945.4:4219410 = 1; NC_002945.4:1254487 = 1; and NC_002945.4:1622803 = 1) and seven clades included human isolates (NC_002945.4:389472 = 8; NC_002945.4:2778919 = 2; NC_002945.4:4219410 = 9; NC_002945.4:1295549 = 2; NC_002945.4:1790349 = 2; NC_002945.4:2232592; and NC_002945.4:1622803 = 2), one of which does not include any isolates from this study.

In general, all the cheese and human isolates had genotypes that were also found in cattle. On average, human and/or cheese isolates were within 8.45 (range 0–17) and 5.8 SNPs (range 0–15), respectively, from cattle isolates (Figure 3, gray shaded clusters). Consequently, nine out of 11 of the cheese isolates were ≤10 SNPs from a cattle isolate from a BCA dairy and this was also true for 11 out of 26 of the human isolates. Within the global context of the *M. bovis* phylogeny, most of the isolates belonged to the European 1 Clonal Complex (Eu1) and only isolates from clade NC_002945.4:1254487 belonged to the European 2 Clonal Complex (Eu2).

**Figure 4:**
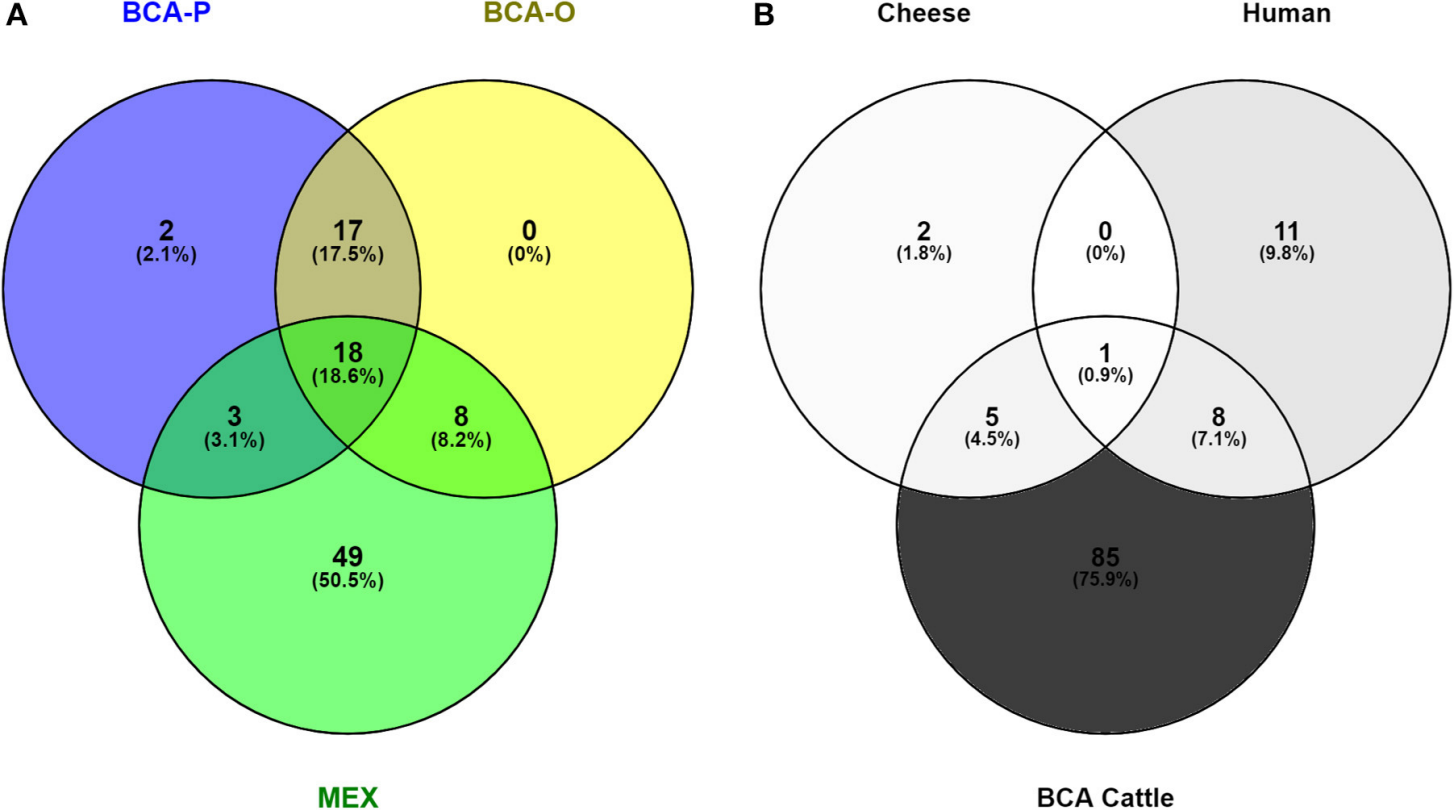
Venn diagrams representing comparisons of *M. bovis* genotypes **(A)** identified in Baja California in this study (BCA-P) and previously (BCA-O, MEX) **(B)** from cattle, humans and cheese.

To determine how much of the diversity of *M. bovis* in BCA has been captured, the genotypes identified in this study were compared to those from previous reports (Figure 4A). From a total of 97 SNP clusters, only two were exclusive to this study (BCA-P), while none were exclusive to what was previously reported in other studies (BCA-O). In total, 19 SNP clusters were identified as specific to the BCA region, while 29 were common to other regions in Mexico (MEX). A total of 49 SNP clusters were found for other regions of Mexico only and not in BCA. Additionally, a comparison between the genotypes isolated from cheese, humans and cattle revealed that one was common to all three sources, five were common to both cattle and cheese and eight were common to cattle and humans (Figure 4B). The larger proportion of SNP clusters (75.9%) belonged only to cattle. A list of the SNP clusters identified in this study can be consulted in Supplementary File 3.

### Putative Transmission Among Dairies

A total of 64 isolates were found that had a pairwise SNP distance of ≤3 SNP to at least one other isolate and the dairies of origin were identified (Table 4). Thirty-three out of the total 61 dairies, which represents 54% of the dairies included in this study, were found to have very closely related isolates, thereby suggesting epidemiological associations between dairies. Most of these associations involved only two dairies and in two instances there were three dairies involved. Moreover, nine pairs of these closely related isolates had identical SNP profiles. Dairy001 was found to have the most associations (*n* = 8), four of which involved isolates with identical SNP profiles (Supplementary File 2). Dairy039 followed closely with a total of six associations, while Dairies 004, 025 and 037 had four each. The rest had only one or two associations.

**Table 4:** Possible epidemiological associations between BCA dairies based on isolates with a difference of ≤3 SNP.

	**Dairies with closely related isolates (≤3 SNP)**	**Total matches within 3 SNPs**	**Total genomes per dairy**
Dairy001*	Dairy003*, Dairy004, Dairy005*, Dairy007*, Dairy011, Dairy047, Dairy050*, Dairy057	8	29
Dairy002	Dairy062	1	2
Dairy003	Dairy001, Dairy004, Dairy025	3	3
Dairy004	Dairy001, Dairy003, Dairy015, Dairy037	4	22
Dairy005*	Dairy001*, Dairy014*	2	7
Diary006*	Dairy064*	1	12
Dairy007*	Dairy001*	1	6
Dairy009	Dairy039, Dairy053	2	7
Dairy010	Dairy037	1	3
Dairy011	Dairy001, Dairy039	2	3
Dairy012	Dairy028, Dairy044	2	5
Dairy014	Dairy005	1	6
Dairy015	Dairy004, Dairy026	2	19
Dairy016	Dairy039	1	8
Dairy023	Dairy025	1	7
Dairy025*	Dairy003, Dairy023, Dairy039*, Dairy040	4	23
Dairy026	Dairy015, Dairy037	2	4
Dairy028*	Dairy012*	1	3
Dairy029*	Dairy037*	1	3
Dairy033	Dairy041	1	8
Dairy037*	Dairy004, Dairy010, Dairy026, Dairy029*	4	18
Dairy039	Diary006, Dairy009, Dairy011, Dairy016, Dairy025, Dairy053	6	24
Dairy040	Dairy025	1	9
Dairy041	Dairy033	1	1
Dairy044	Dairy012	1	11
Dairy047	Dairy001	1	11
Dairy050	Dairy001	1	2
Dairy053	Dairy009, Dairy039	2	1
Dairy055	Unknown	1	6
Dairy057	Dairy001	1	1
Dairy062	Dairy002	1	1
Dairy064*	Diary006*	1	4
Unknown	Dairy055	1	4

*Total associations found for each dairy and total number of high-quality genomes for the specific dairy are indicated. An asterisk indicates associations based on a 0 SNP difference (identical SNP profiles)*.

**Figure 5:**
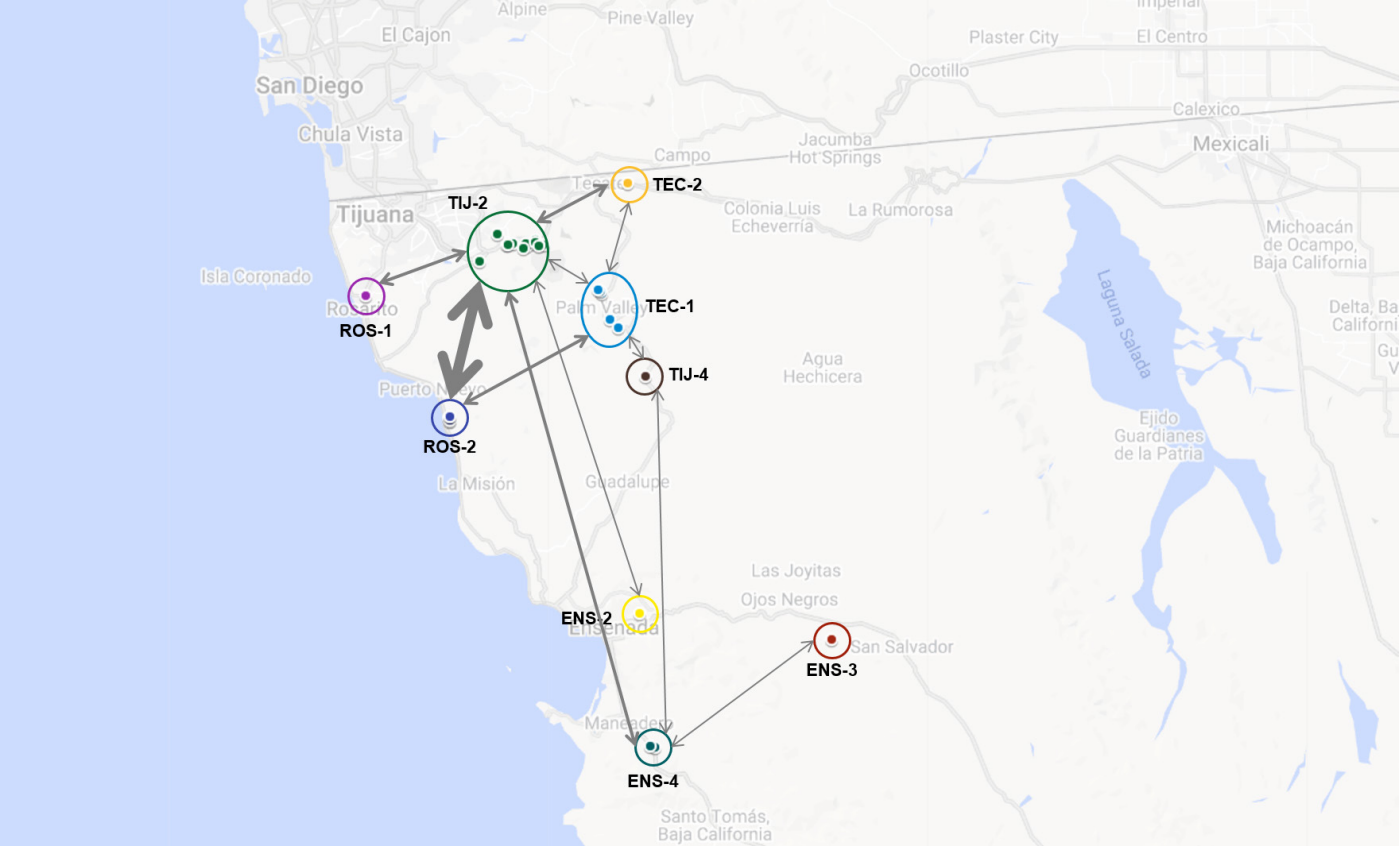
Putative interaction among dairies based on the identification of one or more shared genotypes. Dairies' clusters are indicated and labeled accordingly (ROS-1, ROS-2, TIJ-2, TIJ-4, TEC-1, TEC-2, ENS-2, ENS-3 and ENS-4). Bidirectional arrows are indicative of shared genotypes between dairy clusters and thickness is directly related to the number of shared genotypes (the thicker the arrow, the more shared genotypes). Map was built using GoogleMaps.

These dairies belonged to nine of the 13 previously defined dairy clusters: ROS-1, ROS-2, TIJ-2, TIJ-4, TEC-1, TEC-2, ENS-2, ENS-3 and ENS-4. The frequency at which genotypes were common to two or more dairy clusters is represented in Figure 5. At least one genotype was common to all clusters and each cluster had at least one genotype in common with another cluster. Cluster TIJ-2 had SNP clusters in common with at least six other clusters; of these, ROS-2 had the most shared SNP clusters. Cluster TEC-1 had genotypes in common with at least four other clusters (TIJ-2, TIJ-4, TEC-2 and ROS-2). The rest of the clusters shared genotypes with at least two others and three clusters (ENS-2, ENS-3 and ROS-1) only shared genotype(s) with one other cluster.

## Discussion

The state of Baja California borders the United States, more specifically the state of California, and contains San Ysidro, the busiest border port in the world. Consequently, a high level of cooperation between the governments of Mexico and the US in both human and animal disease surveillance is necessary to address the transmission of the disease between animals, dairy products or people. Tuberculosis is particularly problematic as BCA is one of the states with the highest incidence of TB in cattle and humans in Mexico (10, 45). This study was carried out as a binational effort between the US and Mexico to use WGS to characterize the bTB genotypes circulating in BCA, determine the role of fresh cheese from the region as a potential source of infection to humans and compare the genotypes identified here to those previously reported in the region, Mexico and the US.

Overall, the detection of *M. bovis* from granulomatous tissue at abattoirs in BCA was high, with 84% from histology/culture and 85% from direct PCR/culture. In contrast, the bordering Mexican State of Sonora, which has strict controls to prevent cattle movement from BCA, has an *M. bovis* detection rate of ~1.5% from granulomatous lesions sampled in abattoirs (46). In this study, histology and direct PCR performed nearly the same as culture, which is regarded as the gold standard. Only six and 13 culture positive samples were negative by direct PCR and histology, respectively. Several factors can affect the sensitivity of histopathologic diagnosis, including the multifocal distribution of small granulomas, which may prevent histopathologic identification. On the other hand, 20 and nine samples were negative to culture, but detected by the direct PCR and histology. For PCR, this could reflect amplification of mycobacterial DNA from non-viable organisms; and the absence of acid-fast bacteria upon histological examination may be because of degradation of bacteria or scarcity of bacteria in lesions. Nonetheless, correlation between diagnostic tests (culture vs. PCR and culture vs. histology) was substantial based on kappa coefficients of 0.6774 and 0.7727, respectively.

Genotype characterization of pathogens is essential for disease surveillance, epidemiology and the development of proper control strategies. Previous studies on the diversity of *M. bovis* in cattle in BCA used spoligotyping and VNTR (47, 48) and one study also used WGS (11) to characterize the strains in the region. In comparison to (11), which evaluated 155 *M. bovis* WGS from BCA cattle, our study, which contained 338 WGS, only identified two additional groups, for a total of 10 main *M. bovis* genetic groups circulating in the region (Figure 4A). Overall, 726 WGS sequences, including cattle and cheese have been collected from this region, and based on the significant overlap, we suggest that the genetic diversity of *M. bovis* in BCA is now well-represented. In comparison to other Mexican states, about 30% of the genotypes were common to both BCA and the rest of the country, 20% were exclusive to BCA and 50% were exclusive to the rest of Mexico (Figure 4A). Due to its proximity to the US, it is possible that heifers that were historically imported into BCA may have introduced the disease, as the genotypes found here coincided with those once dominant in the US cattle population (29). Current normativity in Mexico restricts the movement of animals from high-prevalence to low-to-zero prevalence regions, which may explain why the genotypes exclusive to BCA have not been spread. In this regard, the state of Sonora, which separates BCA from the rest of Mexico, is classified as “Advanced Modified Accredited” due to the extremely low prevalence, which may act as a deterrent (or geographical barrier) against the movement of dairy cattle to-and-from BCA and the rest of Mexico through this region (49).

The recovery of *M. bovis* from 2.5% of cheese samples collected from local markets is startling and has far reaching consequences. This suggests that routine consumption of fresh cheese by the local population on both sides of the boarder will likely result in the exposure of infectious tuberculous bacteria by most regular consumers of this product over time. Such a claim does require a high level of evidence and merits further discussion. Supplementary File 6 contains in-depth details of collection date, type of cheese, municipality and store code. While some stores had multiple culture positive samples, all isolates recovered had different WGS profiles, and were collected at different times. Also of note, the isolates recovered from cheese samples most closely matched the diaries located within the same municipality.

Isolation of *M. bovis* from raw milk and cheese is known to present complications due to their complex matrix (high protein and lipid components), as well-susceptibility to contamination by background microflora (50). NVSL has extensive experience developing methods to improve the rate of recovery from these difficult sample types. A contamination rate between 5 and 10%, and a 9.6% recovery rate of acid-fast bacteria suggests decontamination was optimal. Despite this, it is likely that recovery of *M. bovis* does not fully capture the overall prevalence of cheese containing infectious *M. bovis*. This is indicated by the total PCR positivity rate of 6% (19/314). However, because PCR only detects the presence of DNA, the conservative approach is to focus on the samples proven to have live bacteria.

Previous studies have established a relationship between *M. bovis* genotypes found in cattle and humans (51) and positive correlations between the consumption of Mexican fresh cheese and cases of zoonotic tuberculosis (20, 52, 53). Through WGS, this study was able to find a direct relationship (transmission clusters) between *M. bovis* infected dairy cattle in BCA, fresh cheese that originated from the same region, *M. bovis* isolates from humans also from BCA and sporadic dairy cattle in California (Figure 3). Only one previous study, also performed in BCA, used WGS to determine relationships between cases of zoonotic tuberculosis and cattle from the region, also finding positive results (11). The fact that the cheese contains *M. bovis* from the local dairy cattle suggests it is in fact being made with unpasteurized milk from these infected herds. The official norm NOM-243-SSA1-2010 states that milk destined for human consumption is required to undergo “a thermal treatment of a determined time and temperature that guarantees its innocuity,” such as boiling, pasteurization, ultra-pasteurization, sterilization or dehydration, but it exempts the milk that is used for making cheese “whose typical characteristics may not allow it to be made from milk that has undergone thermal treatment” (54). Additionally, NOM-031-ZOO-1995, which is the official norm for the control of bTB in Mexico, states that only 50% of the total national milk production is pasteurized and the rest is consumed [as raw milk] or transformed into dairy products. Consequently, the threat of raw [unpasteurized] milk and its derivatives, possibly from bTB infected cattle, is apparent. Fresh cheeses are a staple food in Mexican households, making them an important source of infection to humans. If pasteurization is not a viable option due to the intrinsic organoleptic characteristics of these types of cheese, cheese-makers must make sure that the raw milk they use comes from healthy animals as to guarantee the innocuity of the product, as stipulated in the official norm for the sanitary requirements of milk and its derivatives (54). In addition to this, education to the public regarding the risks of consuming unpasteurized dairy products could have an impact on their decision to continue to consume these products (55, 56).

For bTB control programs to be successful, strict quarantine measures and animal movement restrictions of infected animals are key. In this study, WGS of *M. bovis* isolates obtained from different dairies throughout the area of study revealed that at least 50% of these dairies were identified with the same SNP clusters or very closely related SNP clusters. This is indicative of herd-to-herd transmission either through the exchange of infected animals or by the acquisition of animals from a same infected source, among other possible causes (57–59). For this study, many dairy owners reported no testing was routinely performed (such as tuberculin skin test for bTB) when introducing new cattle into their herd. In Mexico, the bTB National Program implemented in 1995 has had great success in the beef cattle sector, with most of the focus being on cattle for export to the US. Unfortunately, due to the characteristics of the dairy production systems (longer life/production cycle, higher density of animals, cost of replacement animals, etc.) and relaxed herd management practices (such as lack of testing for newly introduced animals), as well as no compensation of culled animals for farmers, it has been more difficult to reduce the prevalence in dairy regions, where it has been reported at up to 16% (60). Based on experience of the authors, there is a strong and widespread misconception amongst dairy farmers regarding the risk of bTB to human health: because they believe that the milk will ultimately be pasteurized, they don't consider the status of bTB in their herd to be critical.

Another important aspect observed in this study regarding the burden of infection in these BCA dairies is the presence of mixed infections. A mixed infection refers to the simultaneous presence of multiple strains (i.e., genotypes, variants, etc.) of the same pathogen in an individual host. In this study, based on the identification of 20 isolates that presented a high proportion of ambiguous (heterozygous) SNP calls (>50%), nearly 6% of the infected animals had a mixed infection. The high number of heterogenous SNPs point to clearly distinct DNA fingerprints, which support co-infection with different genotypes acquired either at a single point in time or as separate events. This could be indicative of multiple introductions of bTB into the diaries and highlights the lack of control in the movement of animals in the region. Previous studies in *M. tuberculosis* have also found mixed infections in humans more frequently in high-TB burden regions (61). Though a well-studied occurrence in humans, information on identification of mixed *M. bovis* infections in cattle through WGS is scarce; thus, the results shown here may be useful for future comparisons.

A limitation of the study was that sampling only focused on granulomatous lesions observed at carcass inspection. This may have led to the exclusion of animals with an early stage of infection and thus an underestimation of further micro diversity within the herds. However, the large number of animals sampled and the long-term sampling period, as well as the comparison to previously published genomes, support the overall diversity of *M. bovis* seen in the region. Another limitation of the study was the lack of *M. bovis* isolates recovered from humans. In Mexico, mycobacterial culture is not commonly performed in humans, as diagnosis and treatments are initiated using acid fast staining of sputum and PCR. Typically, only cases refractory to treatment are cultured and further characterized. A better understanding of the TB strains infecting humans in the region is needed.

## Conclusions

Despite the well-managed dairy production of this region, a high proportion (93.5%) of the dairies sampled was found infected with bovine tuberculosis. WGS provided evidence of ongoing local transmission of *M. bovis* among these dairies as several of them shared at least one genotype with at least one other dairy in the region. This study was successful at characterizing the diversity of *M. bovis* circulating in the region. This will allow future studies to evaluate the regional and global spread of these genotypes in humans and animals, allowing for a coordinated One Health approach to be used in animal and human TB elimination programs.

## Data Availability Statement

The datasets generated for this study can be found in online repositories. The names of the repository/repositories and accession number(s) can be found in the article/Supplementary Material.

## Ethics Statement

Ethical review and approval was not required for the animal study because all samples were collected as part of authorized regulatory surveillance (NOM-031-ZOO-1995) on animals after harvesting. Written informed consent for participation was not obtained from the owners because all samples were collected as part of authorized regulatory surveillance.

## Author Contributions

SR-A, AP, RM, and EF conceived and designed the study. ED, ER, and KS collected the samples. All authors analyzed the data, wrote, and reviewed the manuscript.

## Conflict of Interest

The authors declare that the research was conducted in the absence of any commercial or financial relationships that could be construed as a potential conflict of interest.

## Publisher's Note

All claims expressed in this article are solely those of the authors and do not necessarily represent those of their affiliated organizations, or those of the publisher, the editors and the reviewers. Any product that may be evaluated in this article, or claim that may be made by its manufacturer, is not guaranteed or endorsed by the publisher.

## Supplementary Material

Supplementary File 1 Total dairies and dairy-clusters included in this study.

Supplementary File 2 SNP tables for the 10 clades associated to *M. bovis* isolates obtained from dairy cattle, humans and cheese in Baja California.

Supplementary File 3 List of *M. bovis* SNP clusters (genotypes) identified in BCA dairies.

Supplementary File 4 List of accessions retrieved from NCBI's Sequence Read Archive.

Supplementary File 5 Sequencing metrics for the genomes obtained in this study.

Supplementary File 6 Metadata and results associated to the cheese samples obtained from Baja California Mexico.
